# Recent Advances in the Development of In Situ Gelling Drug Delivery Systems for Non-Parenteral Administration Routes

**DOI:** 10.3390/pharmaceutics12090859

**Published:** 2020-09-10

**Authors:** Barbara Vigani, Silvia Rossi, Giuseppina Sandri, Maria Cristina Bonferoni, Carla M. Caramella, Franca Ferrari

**Affiliations:** Department of Drug Sciences, University of Pavia, V.le Taramelli 12, 27100 Pavia, Italy; barbara.vigani@unipv.it (B.V.); giuseppina.sandri@unipv.it (G.S.); cbonferoni@unipv.it (M.C.B.); carla.caramella@unipv.it (C.M.C.); franca.ferrari@unipv.it (F.F.)

**Keywords:** in situ gelling systems, drug delivery, polymers, thermo-sensitive systems, ion-sensitive systems, pH-sensitive systems, solid inserts, mucoadhesion, non-parenteral administration routes

## Abstract

In situ gelling drug delivery systems have gained enormous attention over the last decade. They are in a sol-state before administration, and they are capable of forming gels in response to different endogenous stimuli, such as temperature increase, pH change and the presence of ions. Such systems can be administered through different routes, to achieve local or systemic drug delivery and can also be successfully used as vehicles for drug-loaded nano- and microparticles. Natural, synthetic and/or semi-synthetic polymers with in situ gelling behavior can be used alone, or in combination, for the preparation of such systems; the association with mucoadhesive polymers is highly desirable in order to further prolong the residence time at the site of action/absorption. In situ gelling systems include also solid polymeric formulations, generally obtained by freeze-drying, which, after contact with biological fluids, undergo a fast hydration with the formation of a gel able to release the drug loaded in a controlled manner. This review provides an overview of the in situ gelling drug delivery systems developed in the last 10 years for non-parenteral administration routes, such as ocular, nasal, buccal, gastrointestinal, vaginal and intravesical ones, with a special focus on formulation composition, polymer gelation mechanism and in vitro release studies.

## 1. Introduction

In the last decade, the development of in situ gelling drug delivery systems has gained an increasing attention in the scientific community. The majority of these systems presents the peculiarity to be in a sol-state before administration and to undergo gelation into the body. As a consequence, they are characterized by ease of administration, prolonged residence time and sustained drug release at the administration site, with a reduction in administration frequency and an improvement in patient compliance and comfort. Among the reasons for the great success of these formulations, there is the fact that they can be administered through various routes to achieve a local or a systemic effect of the drug loaded. Moreover, they can be successfully used as vehicles for nano- and micro-drug-delivery systems [1,2].

Different are the factors that can trigger the sol–gel transition, such as temperature increase, pH change and presence of ions. Thermo-sensitive in situ gelling systems are in a sol-state at room temperature and are subjected to a sol–gel transition at temperature values close to the physiological one (32–37 °C, depending on the administration site). The mechanism of phase transition is based on a marked change in aqueous solubility of polymers that is characterized by the presence in their structure of hydrophobic and hydrophilic groups. An increase of temperature produces a re-arrangement of polymer–water interactions that, in turn, is responsible for a fast dehydration of the solvated polymer chains and for polymer precipitation. Amphiphilic polymers, which form self-assembled micelles in water, for concentrations higher than the critical micellar concentration, undergo gelation upon increasing temperature. For temperatures higher than the critical micellar temperature, an ordered packaging of the micelles occurs, resulting in gelation [2,3,4]. Among thermo-sensitive polymers, poly(*N*-isopropylacrilamide) (PNIPAM), poloxamers (Pluronic^®^) and cellulose derivatives are the most used in drug delivery.

It is well-known that some anionic polysaccharides (alginate (ALG), gellan gum (GG) and pectin (PEC)) are ion-sensitive polymers [5,6,7,8,9]; they are cross-linked by some monovalent (Na^+^) and/or divalent (Mg^2+^ and Ca^2+^) cations present in different physiological fluids, such as saliva, tears, nasal fluid, etc. The cross-link mechanism produces a sol–gel transition, with the formation of a strong gel. The cation type and concentration are related to cross-linked polymer viscosity and sol–gel transition rate [10].

The pH-sensitive polyelectrolyte polymers are characterized by the presence in their structure of ionizable weakly acidic (carboxylic or phosphoric) or weakly basic (ammonium) groups. Depending on polymer pka, a pH variation produces changes in the ionization state and, consequently, in polymer conformation and solubility that result in gelation. Not only polymer molecular weight (MW) but also temperature and ionic strength of the physiological medium can play a key role. The most widely investigated pH-sensitive polymers are polyacrylic acid (PAA) and chitosan [10,11].

In the case of application on a mucosa, it is desirable that in situ gelling drug-delivery systems possess mucoadhesive properties; that is the capability to interact with mucus and, thus, to further prolong the formulation residence time [3]. A recent novel strategy to achieve a prolonged drug permanence into the administration site and an enhanced drug diffusion across the mucus is represented by dual-functioning formulations composed of nanosystems (NSs) loaded into a mucoadhesive in situ gelling vehicle, able to release NSs after administration [12]. The mucoadhesive in situ gelling vehicle has the function to create an intimate contact with the mucus, providing a greater retention of the loaded NSs at the administration site, whereas the NSs do not bind to mucus components but should be able to cross the mucus barrier, reaching the mucosal surface that represents their site of action/absorption.

With the term “in situ gelling drug-delivery systems” are also indicated solid formulations (i.e., polymeric matrices, films, etc.) that, in presence of biological fluids, undergo a fast hydration, with the formation of a gel able to release the drug loaded in a controlled manner.

This review provides an overview of the advances in the development of in situ gelling drug-delivery systems over the past decade, for non-parenteral administration routes, such as ocular, nasal, buccal, gastrointestinal, vaginal and intravesical ones, taking into account the eventual synergism between gelation and mucoadhesion on the formulation performance. A special focus on formulation composition, polymer gelation mechanism and in vitro release studies is given.

## 2. Ocular Route

The peculiar properties of ocular cavity and its effective clearance mechanisms make the ocular administration of drugs a difficult target with a low therapeutic response. The new generation of ophthalmic formulations has the task of improving the availability of drugs administered by the ocular route and, therefore, their therapeutic efficacy. This can be achieved with the use of in situ gelling formulations that provide an increase in the formulation pre-corneal residence time and, thus, the attainment of an optimal drug concentration at the target site [10].

Such in situ gelling systems, in a sol-state before administration, undergo phase-transition-forming viscoelastic gels in response to one or more environmental stimuli, such as temperature, ions present in the tear fluid and pH [13]. Following topical application, the formation of a gel in the conjunctival cul-de-sac provides sustained release of the loaded drug/s, in order to ensure a prolonged therapeutic effect, reducing dosing regimen and, thus, improving patient compliance. Polymers, generally used for the manufacturing of such systems, are biocompatible, well tolerable and preferably mucoadhesive. Moreover, a pseudoplastic behavior is desirable: It could avoid a painful blink, since it guarantees a reduction in the viscosity of the administered polymeric solution with increasing shear rate [14].

In situ gelling systems can also be used as vehicles of drug-loaded NSs, which are responsible for an improvement in both solubility and corneal permeability of drugs characterized by a poor ocular bioavailability. The incorporation of colloids, generally exhibiting low viscosity, into semi-solid formulations increases their retention time on the ocular surface [15].

Solid in situ gelling formulations, such as polymeric films [16] and electrospun nanofibers [17], have also been proposed for the treatment of topical ocular diseases, even if they currently play only a marginal role as ophthalmic drug-delivery systems. In comparison with liquid formulations, solid systems have the following potential benefits: (i) they can be easily handled and self-applied by the patient, (ii) they are characterized by a better storage stability and (iii) they guarantee a higher ocular drug availability/bioavailability. After ocular application, solid systems quickly hydrate and dissolve, forming a transparent film that is more resistant to clearance mechanisms.

In the following paragraphs, the in situ gelling formulations developed, in the last decade, for the treatment of glaucoma and other ocular disorders are considered.

Glaucoma is the most prevalent ocular disorder, as well as one of the leading causes of irreversible blindness in the world. Such an ophthalmic pathological condition, resulting from an imbalance between aqueous humor drainage and secretion, is accompanied by an increase in intraocular pressure (IOP) that is generally responsible for a damage of the optic nerve and retinal nerve fiber layer. The treatment of glaucoma may include surgery/laser surgery and/or the administration, generally as eye drops, of several therapeutic compounds, such prostaglandin analogues, beta-blockers, cholinergic agonists (i.e., pilocarpine (PIL)), carbonic anhydrase inhibitors and α2-agonists.

In an attempt to overcome conventional ophthalmic dosage form (i.e., eye drops) shortcomings, in situ gelling systems have been proposed, and many papers dealing with thermo-sensitive formulations have been published in the last 10 years.

In 2013, Lai and colleagues have designed a biodegradable thermo-sensitive system, based on gelatin-*graft*-poly(*N*-isopropylacrylamide) (PNIPAAm) (GN) copolymer, for the intracameral administration of PIL in the treatment of glaucoma. Various GN copolymers were synthetized by grafting carboxylic-terminated PNIPAAm with different MWs onto the aminated gelatin, using carbodiimide chemistry. The authors highlighted that the mercaptoacetic acid (MAA)/NIPAAm molar ratio, on which PNIPAAm-COOH MW depended, influenced the performance of PIL-loaded thermo-sensitive systems: In particular, the use of carboxylic end-capped PNIPAAm with high MW was responsible for a lower thermal phase transition temperature and a slower degradation rate with respect to low MW counterparts. With an increase in PNIPAAm-COOH MW, the drug encapsulation efficiency was enhanced due to the fast temperature-triggered PIL capture, while the access of the proteases to the gelatin active sites was limited, thereby leading to a progressive biopolymer network degradation, determining a controlled drug release [18]. A few years later, it has been demonstrated that the performance of in situ gelling GN copolymers was strongly influenced also by the Bloom number of gelatin, which represents an important physicochemical parameter reflecting imino acid and triple-helix contents in the biodegradable polymer. In particular, Chou and colleagues highlighted that the increase in the Bloom index of gelatin resulted in a lower GN phase transition temperature, thus ensuring a high PIL encapsulation. Moreover, a decrease of GN copolymer degradation rate was observed when increasing the Bloom number of gelatin: In vitro results demonstrated that the use of gelatin with high Bloom index stabilized and reinforced the structure of the resulting hydrogel, while improving its resistance to enzymatic activity. A strong dependence of PIL release profile on GN swelling degree and degradability was in vitro demonstrated. Such observation was confirmed also in a glaucomatous rabbit model: The intracameral injection of GN copolymer that was synthetized from gelatin with a high Bloom number ensured a PIL concentration equal to 32.1 ± 1.3 μg/mL, up to 14 days, suggesting enhanced antiglaucoma efficacy [19].

The abovementioned PIL-loaded biodegradable thermo-sensitive GN copolymer was functionalized with antioxidant gallic acid (GA), in order to produce a multifunctional antiglaucoma drug-delivery system with cytoprotective potential against hydrogen peroxide-induced oxidative stress. The synthetized GNGA polymer, characterized by appropriate phase transition temperature and degradation rate, has proven to be suitable for the production of an injectable bioerodible depot for minimally invasive PIL delivery to the ocular anterior chamber. In vivo studies in a rabbit model pointed out positive responses upon intracameral PIL-loaded GNGA injections [20].

In an attempt to produce a long-lasting in situ gelling system for intracameral PIL injection, the same authors proposed the use of chitosan (CS) as an alternative biopolymer to gelatin. In particular, CS-*graft*-PNIPAAm (CS-PN) copolymers, synthetized by grafting PNIPAAm-COOH onto the CS by using carbodiimide chemistry, were characterized by a lower in vitro degradation rate than gelatin-*graft*-PNIPAAm. The slow degradation of CS-PN biodegradable thermogel was responsible for the delayed PIL release, thus ensuring prolonged antiglaucoma effects in a rabbit model during 42 days of the study [21].

**Figure 1:**
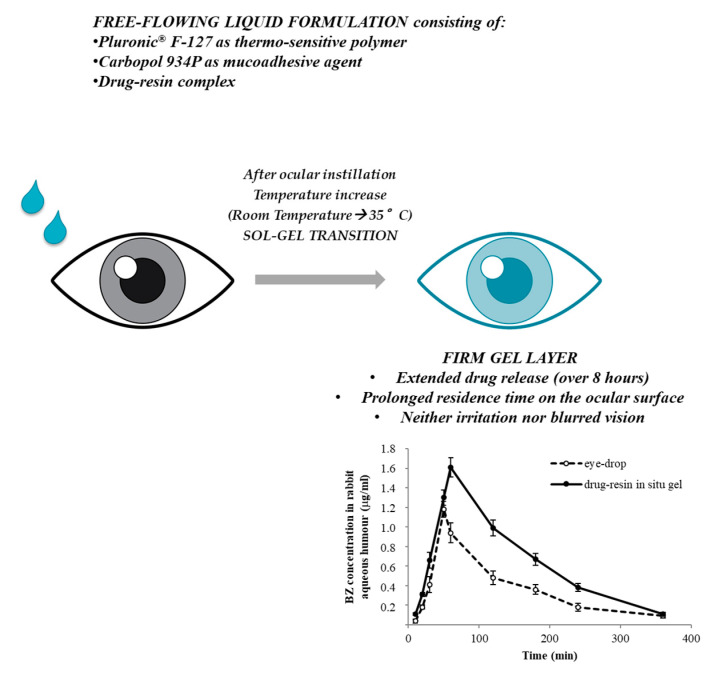
Drug–resin thermo-sensitive in situ gelling system for ophthalmic use: After instillation, an increase in temperature is responsible for the transition of the polymeric liquid formulation loaded with brinzolamide (BZ) into a mucoadhesive gel layer on the ocular surface. The graph represents the concentration–time profiles of BZ in the rabbit aqueous humor: BZ amount in the aqueous humor is significantly higher when BZ is instilled as drug–resin in situ gel than as eye drops. Such results demonstrate that the drug–resin in situ gel is responsible for a higher BZ absorption into the eye: the formation of a gel in the conjunctival cul-de-sac guarantees a prolonged residence time in the pre-corneal area and provides sustained BZ release (Adapted from [22], J-STAGE, 2014).

Li and colleagues combined, for the first time, an ion-exchange resin (Amberlite IRP-69) and a thermo-sensitive in situ gelling system for the brinzolamide (BZ) intracameral administration [22]. The free-flowing liquid formulation, which consisted of poloxamer (Pluronic^®^ F-127) and polyacrylic acid (carbopol 934P (C934P)), was easily instilled in the eye, without causing blurred vision or irritation, and turned into a firm gel under physiological conditions (35 °C), prolonging pre-corneal residence time and improving ocular bioavailability. In particular, the BZ release from drug–resin in situ gel into artificial tears occurred over a period of 8 h: the sustained release mechanism of drug–resin complex, able to exchange drug with the endogenous ocular ions, avoided rapid drug elimination from the periocular area, whereas the in situ gelling process increased the contact time of BZ with the eye. Moreover, the pseudoplastic behavior of the in situ gel, whose viscosity drastically decreased as the shear rate increased, was functional for avoiding patient blinking pain. Finally, animal in vivo studies proved that the developed formulation significantly enhanced drug absorption in the aqueous humor, with respect to commercial BZ eye drops (Figure 1).

A similar thermo-sensitive in situ gelling system, consisting of thermo-reversible poloxamer analogs (P407/P188) and bioadhesive polycarbophil, was developed for ophthalmic delivery of betaxolol hydrochloride: In vitro and in vivo studies pointed out that such a formulation could prolong the drug release up to 8 h, enhancing drug bioavailability and significantly reducing IOP [23].

A mucoadhesive ion-sensitive in situ gel containing acetazolamide (AZA)-loaded nanoemulsion (NE) was developed by Morsi and colleagues [24]: The vehicle was composed of 0.3% *w*/*w* GG, an ion responsive anionic polysaccharide, in combination with xanthan gum, hydroxypropyl methylcellulose (HPMC K4 M) or carbopol 940 as mucoadhesive agent. Considering the low AZA ocular bioavailability due to its poor aqueous solubility and low corneal permeability, its loading in an NE has proven to be an ideal strategy for AZA topical delivery. Despite the optimal physicochemical properties and the low surface tension that guaranteed a good spreading on the cornea, NE was characterized by a limited ocular residence time after administration; therefore, AZA-loaded NE was incorporated in GG-based in situ gel. The GG/xanthan gum system showed superiority over GG/HPMC and GG/carbopol; it showed a significantly sustained AZA release, with respect to NE, good stability and mucoadhesive strength. Moreover, it was characterized by a higher therapeutic efficacy than commercial eye drops and oral tablets, ensuring a more prolonged IOP lowering effect [24]. Afterward, GG was used for the preparation of an in situ gelling system for BZ sustained ocular delivery. The GG-based solution could be easily administered dropwise due to its optimal viscosity and rapidly underwent sol–gel transition following the interaction with ions present in simulated tear fluid (STF). The in situ formation of a strong gel allowed a controlled drug release with respect to marketed eye drops (92% of BZ released within 2 h): the higher the GG concentration, the lower the rate of BZ release (40–70% of BZ released within 12 h) [25]. Such results were confirmed by a recent study of Bhalerao et al. (2020), in which the dependence of the BZ release rate on the GG amount contained in the formulation was discussed in-depth. In particular, with an increase in GG concentration from 0.1 to 0.3% *w*/*w*, the time required for a complete drug release in STF was doubled (from 14.91 to 32.12 h). Upon contact with STF, solutions containing higher GG concentrations turned into stiffer gels, which were able to better control the in vitro BZ release with respect to formulations with a lower GG content. The results obtained by an in vivo study carried out on glaucomatous rabbits have confirmed that GG-based in situ gel have potential in improving BZ ocular bioavailability: The gelation of the polymeric solution inside the cul-de-sac of the eye slowed down drug elimination and prolonged its release, thereby extending the duration of IOP reduction [26]. The use of methacrylated GG derivatives was also investigated in the preparation of PIL-loaded in situ gelling mucoadhesive formulations for ocular drug delivery: the methacrylation improved GG mucoadhesive properties, as demonstrated ex vivo by the higher retention time of GG derivatives on bovine conjunctiva with respect to pristine GG. In vivo experiments highlighted that the methacrylation improved the GG mucoadhesiveness, even if the best performance was observed for the polysaccharide with low degree of modification [27].

In addition to glaucoma, many other ocular disorders affecting both anterior and posterior sections of the eye can be treated through the topical ophthalmic administration of drug-loaded in situ gelling systems with better patient compliance.

Cataract surgery, for example, represents the major cause of ocular inflammation, pain and choroidal neovascularization, which are symptoms responsible for a significant reduction in visual function.

In 2014, Yu and colleagues designed injectable polyethylene glycol (PEG) hydrogels for the ocular delivery of Avastin^®^ (Bevacizumab), which is a chimeric anti-Vascular Endothelial Growth Factor (VEGF) antibody that is able to inhibit the corneal neovascularization after frequent administrations. Transparent PEG gels were quickly formed under physiological conditions via thiol–maleimide reaction between aqueous solutions of 4-arm PEG-Mal (10,000 Da) and 4-arm PEG-SH (20,000 Da). By varying 4-arm PEG-SH concentration, the authors could control the hydrogel gelling time. In particular, the presence of high 4-arm PEG-SH amount in the system promoted a more efficient cross-linking reaction that, in turn, was responsible for a quicker in situ gelation. PEG hydrogels showed no cytotoxic effects in vitro and were able to sustain Avastin^®^ release within a period of 14 days [28].

Afterward, some authors engaged in the development of thermo-sensitive in situ gelling systems incorporating dexamethasone (DXM)-loaded lipid nanoparticles (NPs) for the treatment of ocular inflammation: These novel formulations could be easily administered dropwise in a sol-state, transforming into hydrogels when in contact with ocular surface. In the work of Tan et al. (2017), hydroxypropyltrimethyl ammonium chloride chitosan (HACC) was mixed with β-glycerophosphate (GP) in order to obtain a polymeric blend that quickly turned into a biodegradable gel at around 35 °C. In an attempt to improve the therapeutic efficacy of DXM, whose low aqueous solubility was well-known (0.16 mg/mL), Nanostructured Lipid Carriers (NLCs) were used as promising drug delivery carriers and, then, incorporated into the HACC/GP solution. An in vitro release study highlighted that an NLC-HACC/GP in situ gelling system was able to sustain DXM release during the 48 h [15]. Different combinations of poloxamers (P407 and P188) were, instead, used by Wen and colleagues (2018), for the preparation of an ophthalmic thermo-sensitive in situ gel for DXM-loaded lipid NP delivery. The authors pointed out that the system gelation temperature was influenced by both poly(ethylene oxide)/poly(propylene oxide) (PEO/PPO) ratio and poloxamer concentration: Considering the effect of tear dilution, the poloxamer mixture composed of 18% *w*/*w* P407 and 2% *w*/*w* P188 was selected as the most suitable, since it was able to turn into a gel at 33.4 ± 0.2 °C. In vitro and ex vivo studies demonstrated that the incorporation of DXM-loaded NPs into the in situ gel guaranteed a controlled drug release, a prolonged drug retention time on corneal epithelium and an enhanced drug corneal permeability. Finally, a DXM-loaded NPs containing in situ gel, when administered in the eyes of rabbits, possessed a therapeutic efficacy significantly greater than marketed TobraDex eye drops [29]. The same poloxamer mixture (PP, 18% *w*/*w* P407 and 2% *w*/*w* P188) was investigated as thermo-sensitive ophthalmic vehicle for nepafenac-loaded silica NPs (NSiNPs) topical delivery in comparison with a mixture of P407 and chitosan (PC). PC-NSiNPs underwent sol–gel transition at 32 °C, forming a hydrogel in which NSiNPs were homogenously dispersed; the system was characterized by a sustained drug release and higher ex vivo corneal permeation (58.79 μg) than PP-NSiNPs (21.18 μg), probably due to chitosan bioadhesive and penetration-enhancing properties [30]. In the work of Gonzalez-Pizarro et al., P407 alone was used as thermo-reversible polymer for the development of an in situ gelling system in which fluorometholone (FMT)-loaded poly(lactide-*co*-glycolide) PLGA NPs were dispersed. The formulation was in a sol-state at room temperature, allowing an easy administration in eye drop dosage form, while turned into gel at a temperature close to 34 °C. The addition of methylcellulose (MC A4M) was functional to improve the bioadhesion of the system, thereby prolonging its pre-corneal residence time. The thermo-reversible behavior and the presence of MC promoted an increase in system viscosity at corneal temperature, which was responsible for improved FMT ocular bioavailability and anti-inflammatory effect: FMT slow and prolonged release allowed the drug to reach aqueous humor and crystalline [31].

In the same years, Tatke and colleagues have designed a GG-based ion-sensitive in situ gelling system containing triamcinolone acetonide–loaded Solid Lipid Nanoparticles (TA-SLNs). An ex vivo permeation study, performed on the corneas isolated from rabbit whole eyes, demonstrated that TA-SLN in situ gel was characterized by an improved permeability with respect to TA-SLNs: After contact with the ions present in the ocular tear film, the system underwent in situ gelation, enhancing drug absorption. This result could be related to a prolonged TA residence time on the ocular surface and/or in the conjunctiva sac, a sustained TA release and/or a limited pre-corneal TA loss [32]. Moreover, Ranch and colleagues proposed the development of an in situ gel containing GG and C934P, which acted synergistically to promote in situ gelation. The system developed was characterized by several properties (i.e., clarity, pseudoplastic behavior, adhesiveness, etc.), which made it particularly promising as ophthalmic formulation for topical application; finally, the in vivo results highlighted that the drug (olopatadine HCl) persisted in the rabbit tear fluid up to 3 h in comparison to eye drop solution that was removed from the eye within 1 h [33].

Ocular disorders also include corneal neuropathy and keratopathy, which are responsible for different damages to the anterior eye segment structures, such as corneal epithelium fragility, alterations and instability of the tear film (dry eye disease, DED), impaired corneal wound healing process and increased risk of infections.

In 2014, Abdelkader and colleagues proposed a variety of in situ gelling polymeric films as single-dose solid units of naltrexone hydrochloride (NTX) for topical ocular administration; such a strategy was adopted to deal with the poor chemical stability in aqueous solution of NTX, which is an opioid growth factor receptor antagonist used for DED treatment and wound healing application. Fifteen film formulations based on polyvinyl alcohol (PVA) were prepared using the solvent casting method: PVA, a well-known film-forming polymer, was differently combined with MC, carboxymethylcellulose (CMC) and/or ALG, which were selected for their gelling and mucoadhesive properties. Five ocular film formulations were selected for their mucoadhesion (work of adhesion ≥ 60 mN·mm) and malleability (strain ≥ 5%), by which the authors meant the film ability to conform to the curvature of the eye globe, permitting normal eye blinking. According to both the formulation composition and the drug solubility in the polymeric matrix, NTX was completely released from the five ocular films within 3–10 h. The formulation containing PVA, CMC and ALG proved to be characterized by superior mucoadhesive properties, excellent chemical and physical stability as well as minimal conjunctival irritation [16]. In 2016, Soluplus^®^ (polyvinyl caprolactam-polyvinyl acetate-polyethylene glycol copolymer)-based nanomicelles were demonstrated able to enhance the apparent solubility (more than 10-fold than commercially available eye drops), stability and corneal permeability of α-Lipoic acid (ALA), which is an antioxidant compound potentially useful in the treatment of diabetic keratopathy and retinopathy. The thermo-sensitive properties of the aqueous dispersion of ALA-loaded Soluplus^®^ nanomicelles allowed its transition from a free-flowing liquid system to a weak gel at ocular surface temperature; in situ gelation was functional to increase corneal residence time, while improving ALA accumulation into the cornea (ex vivo corneal permeability test). Moreover, ALA-loaded Soluplus^®^ nanomicelles demonstrated stability upon strong dilution in STF, filtration, freeze-drying and reconstitution in aqueous media, proposing as a valid alternative to current ALA formulations [34]. In the context of severe-DED treatment, a novel artificial tear formulation was designed by Acar and colleagues: a liposomal suspension containing phosphatidylcholine, cholesterol, vitamins A and E was diluted with a solution composed of GG, HPMC, osmoprotectants (levocarnitine and trehalose) and electrolytes (NaCl and KCl). The ion-sensitive properties of the formulation due to the presence of GG guaranteed its resistance to the physiological drainage, extending its residence in the eye for the time necessary to restore the tear film and protect the corneal epithelium [35].

In the last 10 years, in situ gelling formulations loaded with antimicrobial and antifungal compounds, as such or encapsulated in lipid/polymeric NSs, have been developed for the treatment of infections associated to corneal ulcers. For this purpose, different thermo-reversible [36,37,38,39], ion-sensitive [40,41,42,43] and/or pH-triggered [44,45] polymers were used, often in association with cellulose derivatives, such as HPMC and MC, as mucoadhesive and viscosity-enhancing agents.

As for commercially available in situ gelling formulations, Timoptic-XE^®^, containing timolol maleate, a non-selective β-adrenergic receptor blocking agent, and based on GG (Gelrile^®^), has been on the market since 1994. AzaSite and Pilopine HS are polyacrylic acid based ophthalmic solutions indicated, respectively, for the treatment of bacterial conjunctivitis and to control intraocular pressure [46].

## 3. Nasal Route

The nasal cavity has been emerged as an attractive route of multi-site targeting for the administration of a wide plethora of drugs, from small compounds to biological macromolecules, including peptides, proteins and vaccines [47].

The nasal route is the natural choice for the topical administration of drugs intended for the treatment of local disorders affecting the nose and the paranasal sinuses, such as allergic or infectious rhinitis, sinusitis, rhino-sinusitis and nasal epithelium lesions [48,49]. In addition, the nasal mucosa represents a non-invasive alternative route for the systemic delivery of drugs with poor bioavailability; in fact, the highly vascularized nasal epithelium has been exploited to achieve a rapid absorption of drugs that usually undergo to an extensive first-pass metabolism and/or gastric degradation after oral administration [49]. Moreover, the nasal route has proven to be beneficial also for delivering drugs to the brain, circumventing the blood–brain barrier (BBB) that restricts the diffusional transport mechanisms of several therapeutic agents after oral or parenteral administration. The nose-to-brain delivery guarantees the direct and rapid transport of drugs from the nasal cavity to the central nervous system (CNS) through the olfactory neuroepithelium [48,50].

Even if the intranasal route offers several advantages in terms of accessibility, efficacy, tolerability and patient compliance, the mucociliary clearance represents the physiological factor mainly involved in the reduction of the drug residence time in the nasal environment. Such a self-clearing mechanism is responsible for the rapid removal of the drug from the nasal cavity, thereby reducing the time needed for the drug to treat the nasal local diseases or to reach the systemic bloodstream or the CNS [47,48,49]. In an attempt to prevent the rapid drainage of drugs, when administered as simple aqueous solutions, and to prolong their residence time in the nasal cavity, a viscosity-enhancing approach has been proposed: nasal in situ gelling formulations seem to be a more effective alternative to nasal liquid ones [47]. Such formulations are easily administered as low viscosity polymeric solutions, ensuring an optimal nasal deposition, and turn into gels upon contact with the mucosa. The sol–gel transition can be induced by different physical or chemical stimuli, in particular temperature, pH and ionic strength: The in vivo formation of a polymeric network prolongs the contact time between the drug and the site of action/absorption and also guarantees a sustained release of pharmaceutical ingredients [51].

Nasal administration of corticosteroids is the first-line therapeutic strategy for the local treatment of nasal inflammatory disorders. Numerous research groups have confirmed the unique benefits of the in situ gelling systems for topical drug delivery into the nasal cavity: the rapid gel formation not only reduces the mucociliary clearance effect but also hinders the permeation of locally acting drugs through the nasal mucosa, thus limiting their systemic absorption [52].

Dukovski and colleagues [53] developed an in situ gelling formulation for corticosteroid delivery in the treatment of chronic rhinosinusitis with nasal polyps. In particular, dexamethasone (DEX)-loaded lipid/ALG nanoparticles (NPs) were dispersed in a PEC solution that could be easily sprayed at the site of inflammation by an appropriate nasal delivery device; the ability of PEC to form gels upon contact with the Ca^2+^ ions present in the nasal mucosa is responsible for the sol–gel transition. The design of such NP-containing in situ gel was functional to achieve a prolonged local anti-inflammatory effect: DEX loading into NPs retarded DEX release from NP-containing gel, since the diffusion of DEX-loaded NPs through PEC gel was hindered by the entanglement of polymeric chains and their possible interaction with NP surface. Another in situ gelling system obtained by the interaction between a polymer and the Ca^2+^ ions was developed by Nižić et al. [52]. The authors prepared a fluticasone-loaded in situ gelling suspension that was optimized in terms of nasal deposition after manual spraying under breathing conditions; the sprayability and the turbinate deposition of the formulation have been considered to be the pivotal factors influencing the outcome of the local treatment. In Nižić’s work, PEC and GG, both known for their ability to interact with divalent cations, were used as gelling agents, while sodium hyaluronate was chosen as bioactive gel-structuring ingredient, contributing to the healing of mucosal surface lesions. The interaction between GG and the ions present in the mucus layer on the nasal mucosa was already investigated in 2009, by Cao and co-workers [51], who developed an in situ gel loaded with mometasone furoate (MF). In this paper, the authors stressed the importance of selecting a gelling-agent concentration that could guarantee both an easy nasal administration and a rapid formation of a gel strong enough to sustain the corticosteroid local release, without dissolving or eroding quickly.

The use of thermo-reversible polymers for the preparation of nasal in situ gelling systems was also exploited by other authors, such as Altuntaş and Yener [54]. In their work, P407 was used in combination with Carbopol^®^ 974P NF, a bioadhesive polymer, in order to produce a thermo-sensitive gel for the prolonged release of MF in the treatment of allergic rhinitis. The poloxamer concentration was optimized so that the sol–gel transition temperature (Tsol–gel) of the in situ gelling system developed was lower than the nasal physiological one. The increase in P407 concentration leads to a decrease of formulation Tsol–gel and, thus, to the rapid formation of a well-structured gel. Altuntaş and Yener have demonstrated that a P407 concentration of 18% *w*/*v* was able to quickly produce a gel at almost 30 °C, while ensuring an accurate drug dosing at room temperature. Pandey et al. have selected a poloxamer concentration equal to 15.5% *w*/*w* as optimal for the preparation of a thermo-sensitive system characterized by a Tsol–gel suitable for nasal administration [55]. The poloxamer was used in combination with bioadhesive polymers, such as HPMC E4M and CS, in order to enhance the adhesion of the gel, loaded with dexamethasone 21-phosphate disodium salt, on the nasal mucosa.

In situ gelling formulations were used not only as corticosteroid delivery systems, but also for the local treatment of nasal wounds. Gholizadeh and co-workers [56] developed a thermo-sensitive and mucoadhesive formulation based on the use of CS that is well-known for its hemostatic and wound healing properties; the addition of GP, a weakly basic organic compound neutralizing the pH of CS solution, was required to obtain a formulation able to undergo a sol–gel transition in response to a temperature shift. Tranexamic acid (TXA), an effective and good tolerable drug commonly used to control bleeding, was loaded into the CS–GP solution that has proven to be in a sol-state at room temperature and capable to form a gel at 32 °C within approximately 5 min, revealing itself suitable for nasal administration.

In the last decade, nasal administration of antiemetic and motion sickness drugs has been exploited to achieve their rapid absorption in the systemic circulation. The highly vascularized and permeable nasal mucosa ensures a fast onset of drug action that is particularly crucial in the management of severe nausea and vomiting, which are often associated with cancer therapy or migraine.

Nasal administration has aroused particular interest in the systemic drug delivery, since it improves patient compliance: Unlike parenteral administration, it is painless and allows self-medication. Moreover, nasal delivery guarantees an accurate and consistent drug dosing, considering that nausea and vomiting are causes of gastric dysmotility and, thus, of significant alterations of drug intestinal absorption after oral administration [49].

Metoclopramide hydrochloride (MCP HCl) is a potent antiemetic compound that, when orally administered, is characterized by both a highly variable bioavailability (from 32% to 98%), due to its extensive first-pass metabolism, and a short half-life requiring three or four administrations a day. MCP HCl intranasal administration has been proposed as an effective alternative. Considering the low MCP HCl permeability across the nasal mucosa and the mucociliary effect, the use of in situ gelling systems seems to be a good formulation strategy to prolong MCP HCl residence time in the nasal cavity. Zaki and colleagues [57] developed a MCP HCl-loaded mucoadhesive in situ gel that contains P407 as the thermo-reversible polymer, polyethylene glycol (PEG) as the release enhancer, and C934P as the mucoadhesive agent. The bioavailability of MCP HCl was investigated in rabbits for the optimized nasal in situ gel in comparison with the oral drug solution and a significant increase in MCP HCl bioavailability from 51.7% (oral drug solution) to 69.1% (nasal in situ gel) was observed. The authors concluded that the in situ gelling formulation developed was able to quickly form a gel after administration and to retain the drug in the nasal cavity for a time long enough to guarantee its absorption across the mucosa.

**Figure 2:**
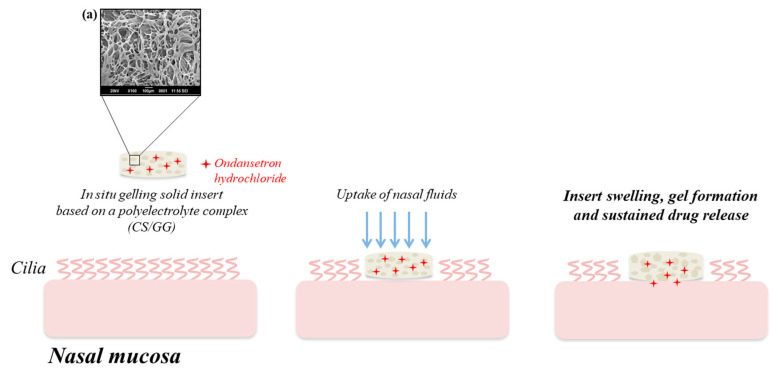
In situ gelation of a solid nasal insert loaded with ondansetron hydrochloride, prepared by freeze-drying of an aqueous polymeric solution consisting of chitosan (CS) and gellan gum (GG); (**a**) scanning electron micrograph of the freeze-dried insert (Adapted from [58], ELSEVIER, 2016). Upon contact with the nasal mucosa, the porous structure of the insert allows rapid hydration of the cross-linked polymeric matrix and the consequent formation of a gel that guarantees a controlled drug release (Adapted with permission from [47], ELSEVIER, 2016).

The intranasal delivery appears an attractive alternative also for the administration of ondansetron hydrochloride (OND), a selective 5-HT3 receptor antagonist used for the prevention of nausea and vomiting after radio- and chemotherapy or surgical operations. In 2016, Sonje and Mahajan [58] developed an OND-loaded in situ gelling nasal insert, prepared by freeze-drying of an aqueous polymeric solution consisting of CS–GG polyelectrolyte complex (Figure 2).

The cross-linking of the cationic CS with the oppositely charged GG allowed the production of a three-dimensional network of polymeric chains in which the drug was dispersed. Nasal inserts are solid dosage forms consisting of sponge-like hydrophilic polymer matrices, which, once administered, uptake the physiological fluids present in the nasal cavity and rapidly form gels from which the drug is released in a controlled manner. The highly porous structure of such insert is responsible for an increased water penetration and, thus, for a greater swelling from which depends both the prolonged nasal residence time and the slow drug release. Animal studies demonstrated that the nasal insert developed by Sonje and Mahajan not only guaranteed an accurate drug dosing, but also enhanced the ondansetron systemic absorption with respect to the oral drug solution [58].

Finally, the nasal administration has been also exploited for the treatment of neurological diseases; several drugs, such as anti-migraine, anti-Parkinson, antidepressant, anti-anxiety, antipsychotic and antiepileptic compounds, have been loaded into both liquid and solid in situ gelling formulations.

For the long-term treatment of psychiatric illness, Luppi and colleagues [59] developed chlorpromazine hydrochloride–loaded nasal inserts, which, upon contact with nasal fluids, rapidly form gels able to prolong the drug permeation across nasal mucosa that is functional to reduce the number of required daily dosing and to eliminate peak-to-valley fluctuations, thus limiting the onset of side effects. Such nasal inserts were obtained by freeze-drying of CS/PEC polyelectrolyte complexes in which the drug was dispersed. The authors highlighted that the polycation/polyanion molar ratio was a crucial factor that influences the insert porosity and, thus, the water permeation. The insert swelling behavior was strictly correlated with the drug release profile, influencing drug bioavailability over time as demonstrated by in vitro permeation studies across sheep nasal mucosa.

In the last decade, several authors have investigated the use of thermo-sensitive formulations for the nasal administration of anti-migraine drugs, such as sumatriptan [60], rizatriptan benzoate [61] and zolmitriptan [62]. Shelke et al. [63] developed an in situ gelling bioadhesive formulation consisting of P407 and C934 for naratriptan hydrochloride (NH) delivery to the brain via the olfactory lobe pathway. Such a formulation was able to form a gel at 29 °C and retain a gel-like structure for 47 s (desirable time), when subjected to a shear stress. In vitro release studies revealed that NH release (more than 65% after 12 h) was controlled by the drug diffusion rate and the relaxation of the polymeric chains. According to the ex vivo experiments, the authors concluded that C934 non only acted as mucoadhesive agent, but also as penetration enhancer, while P407, selected as thermo-reversible polymer, was responsible for a sustained drug release.

A recent investigation conducted by Wavikar and co-workers [64] has explored the potential of a nasal in situ gelling formulation incorporating rivastigmine (RV)-loaded NLCs in the systemic treatment of Alzheimer’s disease. In order to ensure an optimal resistance to mucociliary clearance, GG and poloxamer (Lutrol 127), which are two polymers responsive to different stimuli (ions and temperature, respectively), were used in combination for the preparation of such system. Brain targeting potential of the formulation was assessed by in vivo pharmacokinetic and pharmacodynamic studies: RV concentration in the brain was 1.61 times more when the NLCs were intranasally administered in the in situ gelling system, as compared with intravenously administered ones.

Nose-to-brain delivery was also exploited in the treatment of AIDS dementia complex, a CNS disorder that occurs when human immunodeficiency virus (HIV) enters in the brain tissues; in particular, Ved and Kim [65] prepared an in situ gelling system, based on the use of poloxamer as thermo-reversible agent, for the enhancement of intranasal zidovudine (ZVD) delivery to the brain. The in vivo absorption and brain distribution studies in rabbits revealed that the ZDV concentrations in both the cerebrospinal fluid (CSF) and the brain, achieved after intranasal administration of thermo-sensitive system, were approximately five times greater than those attained after intravenous injection.

## 4. Buccal Route

In the last decade, the administration of in situ gelling systems in the oral cavity has been principally exploited for the local treatment of oral mucositis, appearing as a valuable strategy to control pain, modulate the inflammatory response, enhance the wound healing process and prevent bacterial and fungal infections.

Oral mucositis represents the most common and clinically significant complication of systemic chemotherapy and/or radiation therapy in patients with head and neck carcinoma. Such a pathological condition is generally characterized by a thinning of the oral epithelium that leads to inflammation and ulceration of the mucosa, mostly associated with an intense pain and bleeding. Oral mucositis significantly affects the patient nutritional status and quality of life, sometimes increasing the risk of infections in the oral cavity; in the most severe conditions, clinicians are forced to reduce the dose of chemotherapeutic agent/radiations or to temporary discontinue therapy [66,67].

In the attempt to prevent or reduce the discomfort caused by the ulcerative lesions in the mucosa, the current first-line treatment in most United States hospitals consists of an oral rinse with a solution containing a local anesthetic (e.g., lidocaine). However, pain relief effect is modest and short-term (less than 30 min) probably due to the limited contact of the anesthetic solution with the damaged mucosa. Moreover, repeated rinses throughout the day lead to the numbing of the whole oral mucosa, including healthy regions. Other conventional formulations, such as mouthwashes and polymeric gels, have also been used for the local delivery of anti-inflammatory drugs, healing promoters or antimycotics; anyway, their low residence time in the oral cavity, attributable to the saliva flushing effect and the tongue cleansing action, results in the failure of the therapy [68].

In this context, the use of mucoadhesive in situ gelling systems represents a valid approach to overcome the shortcomings of conventional treatments. After local application on the injured mucosa, the polymeric solution, in response to different stimuli (i.e., temperature variations, presence of divalent ions, etc.), turns into a mucoadhesive gel, thus creating a protective layer on the ulcerative lesions that reduces the risk of microorganism infections. The peculiar design and composition of such systems ensure both a prolonged residence time at the injury site and a drug sustained release, to avoid repeated administrations and to improve patient compliance [69].

In 2010, in a work of ours, we developed a thermo-sensitive mucoadhesive gel obtained by the mixture of trimethyl chitosan (TMC), characterized by both antimicrobial and wound-healing properties, and GP. The gelation of TMC/GP mixture should occur after administration in the oral cavity. It was demonstrated that the increase of temperature (from room to body temperature) produces a strengthening of hydrophobic interactions between chitosan chains and/or a proton transfer from TMC to GP that neutralizes the chitosan derivative and, thus, allows the formation of a physical gel as a result of interchain attractive forces. The TMC/GP system, loaded with benzydamine hydrochloride, was able to control drug release (70% of the drug loaded was released after 5 h) and to withstand the physiological removal mechanisms (40% of the drug loaded remained on the mucosa after 5 h) [70]. A year later, we designed a sprayable thermo-sensitive system, based on P407 and ALG, for the local delivery of platelet lysate (PL), a hemoderivative well-known for its wound healing properties. The formulation developed was characterized by a low viscosity at 8 °C, that was functional for the extemporary PL loading and for spraying, and was able to quickly turned into gel at 34–35 °C [71]. The use of a sprayable in situ gelling formulation, easy to self-apply in any region of the oral cavity, is preferable and improves the effectiveness of the treatment [72].

More recently, we developed an in situ gelling system consisting of κ-carrageenan (κ-CG) and hydroxypropyl cellulose (HPC), which has proven to possess the following features: (i) low viscosity at room temperature, making it suitable for an easy administration; (ii) marked elastic behavior at body temperature, functional to a protective action toward the application site; and, finally, (iii) mucoadhesive properties for a prolonged permanence on the injured mucosa. κ-CG was selected as an ion-sensitive polymer able to interact with saliva ions, HPC was used as mucoadhesive agent and CaCl_2_ was identified as a salt able to enhance the interaction between κ-CG and saliva ions [73]. In a subsequent work, *Hibiscus sabdariffa* extract, which is rich in phytochemicals with antioxidant and anti-inflammatory properties, was loaded in the κ-CG/HPC system previously developed [74].

Other innovative and versatile systems that can be easily applied on the injured oral mucosa are represented by lyophilized wafers, which can be prepared by freeze-drying aqueous solutions of hydrophilic polymers, generally carbohydrates, in order to obtain solid porous matrices with a sponge-like morphology. Sustained drug release can be achieved by using lyophilized cross-linked polymeric hydrogels. After local application, wafers do not dissolve, but gradually imbibe saliva and swell, thus ensuring longer residence time on the ulcerative mucosa. The saliva penetration within the matrix structure is responsible for polymeric chain hydration and relaxation: The loaded drug is released in a controlled manner, by diffusion, through the polymeric network and/or following the erosion of the matrix [75].

**Figure 3:**
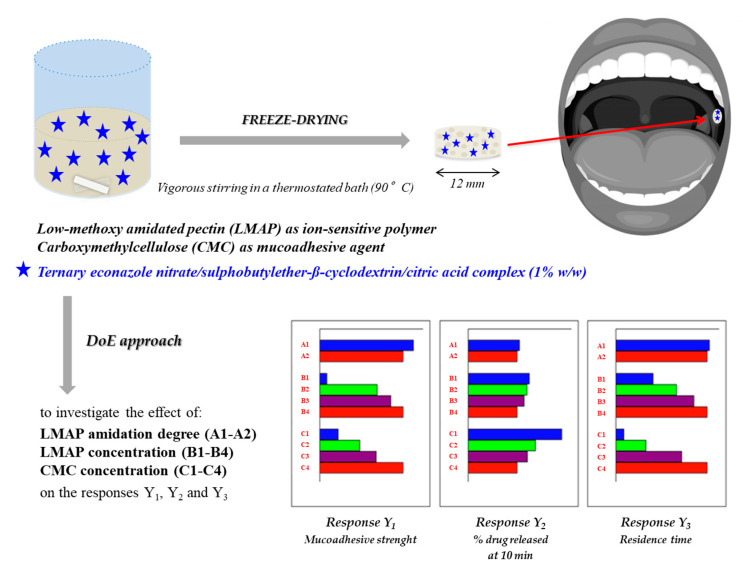
Schematic representation of the preparation method and the application of lyophilized wafers for the local delivery of econazole nitrate in the treatment of oral candidiasis: Low-methyl-ester-amidated pectin (LMAP) is able to gel upon contact with saliva ions, while carboxymethylcellulose (CMC) ensures mucoadhesive properties. In the attempt to optimize the formulation, a DoE (Design of Experiments) approach was used to individuate the factors whose variation could influence the wafer performance in terms of mucoadhesive strength (response Y1), % drug released at 10 min (response Y2) and in situ residence time (response Y3). In a screening design, the authors selected LMAP amidation degree, LMAP and CMC concentrations as the critical independent variables. The variation of LMAP amidation degree (A1–A2) did not influence any of the considered response, while an increase in both LMAP (B1–B4) and CMC (C1–C4) concentrations significantly increased the responses Y1 and Y3. A central composite design was then considered with the aim of optimizing the formulation (Adapted with permission from [77], ELSEVIER, 2015).

In 2012, Shaikh and colleagues demonstrated that aqueous solutions of PEC, after the addition of different salts, such as calcium sulfate (CaSO_4_), barium chloride (BaCl_2_) and zinc sulfate (ZnSO_4_), and subsequent freeze-drying, were able to produce lyophilized cross-linked wafers able to provide a controlled drug release after contact with simulated saliva [76]. Such a peculiar buccal system was also proposed for the treatment of oral candidiasis; in particular, Mura et al. developed a lyophilized wafer, consisting in low-methyl-ester-amidated pectin (LMAP), able to form a gel after contact with saliva, due to the presence of calcium ions, and CMC, for the delivery of econazole nitrate (ECN) in the oral cavity. A DoE (Design of Experiments) approach was used to investigate the effect of LMAP amidation degree, LMAP and CMC concentrations on the wafer performance in terms of mucoadhesive strength, in situ residence time and % drug released (Figure 3). The optimized formulation guaranteed a controlled ECN release (5 mg/h), thus showing an antifungal activity against selected Candida strains in vitro [77].

In situ gelling systems were also developed for intra-pocket drug delivery in the treatment of periodontitis. Periodontitis is an immune-inflammatory condition of the supporting periodontal tissues, principally caused by the ability of some pathogens to colonize the tooth surface and the surrounding areas. The microbial infection triggers an acute inflammatory response that, in turn, is responsible for the destruction of the sub-gingival tissues, the periodontal ligament and the alveolar bone. The progression of such inflammatory reaction leads to gingival recession and then to the formation of periodontal pockets, which provide a favorable environment for the growth and proliferation of some Gram-negative anaerobic bacterial species [78].

The periodontitis management generally requires a deep cleaning procedure (root planing) below the gum line, followed by the intra-pocket administration of antimicrobial and/or anti-inflammatory drugs. Nowadays, several intra-pocket drug delivery systems, such as solutions, gels, fibers, films and inserts, have been investigated and, sometimes, proposed on the market. Nevertheless, such systems show some limitations resulting in the reduced treatment efficacy and/or low patient compliance; for example, in the case of solutions, the drug at the site of infection/inflammation has poor retention, and the drug release rate is not controlled, while, in the case of inserts, there is pain caused by their insertion in the sub-gingival areas [79,80].

**Figure 4:**
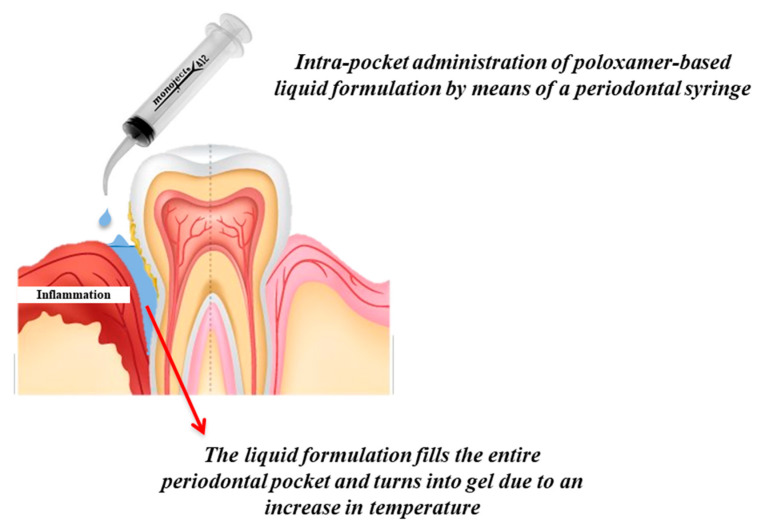
Rationale for the use of thermo-sensitive in situ gelling system for topical intra-pocket delivery of anti-inflammatory and/or antimicrobial compounds in the treatment of periodontitis.

For these reasons, in situ gelling systems, in particular thermo-sensitive ones, have been proposed as valid candidates for local intra-pocket drug delivery. Being fluids before and during administration, they can be easily injected by means of a periodontal syringe, allowing the formulation to get access to the entire pocket; once administered, they undergo sol–gel phase transition resulting in soft but robust gels that are able to prolong the release rate of the drug, residing at the site of action for a desirable period of time (Figure 4) [79,81].

Poloxamer is the thermo-reversible polymer most used for the manufacturing of in situ gelling systems intended for the local treatment of periodontitis. Kassem and colleagues [81] prepared poloxamer-based formulations containing either meloxicam (Mx), an anti-inflammatory drug, or minocycline HCl (MH), an antimicrobial agent. The high poloxamer concentration (35% *w*/*v*) was responsible for the large number and size of polymeric micelles, which, in turn, increased gel rigidity and tortuosity. As a consequence, both Mx and MH were characterized by a prolonged release profile, probably controlled by both diffusion and chain relaxation. Moreover, a clinical evaluation of both formulations was performed on patients suffering from periodontal pockets: The MH-loaded system, which released in vitro about 85% of the loaded drug within three days, showed the best performance, reducing pocket depth and gingival inflammation and increasing alveolar bone density. Some authors have also explored the use of in situ gelling systems, based on poloxamer, for the intra-pocket co-administration of dual antimicrobials in order to extend the antibacterial activity against a broad range of pathogens [82,83].

Afterward, Morelli et al. [79] developed a thermo-sensitive system, based on poloxamer, containing β-cyclodextrin-Jeffamine microparticles for the sustained release of chlorhexidine (CHX), while Beg and co-workers [80] designed a novel poloxamer-based gelling formulation in which PLGA nanoparticles loaded with moxifloxacin (MOX-NPs) were dispersed. In the latter work, in vivo studies revealed that the system developed was able to reside at the periodontal site for a prolonged period of time, guaranteeing an extended MOX release. In particular, once-a-week injection of thermo-gel loaded with MOX-NPs was demonstrated to be more effective than twice-a-day application of a conventional marketed gel.

## 5. Gastrointestinal Route

In the literature of the last 10 years, research works dealing with the development of in situ gelling formulations for local (rectal–colonic) administration of drugs have been reported. Different approaches have been used, including the following: the sol–gel transition was triggered by an increase in ion concentration or temperature or by a change in pH. Some studies have employed multiple approaches, leading to the development of formulations based on mixtures of ion-, temperature- and/or pH-sensitive polymers. Hereafter, we report some significant examples.

Narita et al. investigated the capability of collagen–genipin solutions for endoscopic treatments of gastrointestinal ulcers [84]. Genipin acted as cross-linker. Collagen–genipin solutions were characterized by a low viscosity at 23 °C that allows an easy endoscopic use. At the physiological temperature and in presence of phosphate buffer (pH 7), a gelation occurred with gel deposition on ulcers.

Jensen et al. [85] developed a rectal formulation for the treatment of radiation-induced proctitis (RIP) that is the most common clinical adverse effect of radiotherapy for ovarian, prostate, colon and bladder cancers. The formulation was based on silk-elastin like protein polymer (SELP 815K) containing six repeats of blocks comprised of eight silk-like units, 15 elastin-like units and one lysine-substituted elastin-like unit. When in solution, SELP 816K was able to transient from a low viscosity liquid at room temperature to a gel at physiological temperature. Such formulation was used to release in the rectal cavity a semi-synthetic glycosaminoglycan (GAG) obtained from the sulfation of hyaluronic acid with anti-inflammatory properties. The authors proved in a murine model that the formulation was able to reduce the radiation-induced pain.

Antonino et al. [86] developed an in situ gelling formulation using poloxamer P127, as thermo-reversible polymer, and entrapping budesonide (BUD), a potent corticosteroid used for the treatment of gastrointestinal inflammatory diseases. In vivo studies performed in a murine model of intestinal mucositis proved that the formulation was effective in the treatment of inflammatory injuries of the intestinal mucosa.

An in situ thermo-gelling hydrogel based on methoxy poly(ethylene glycol)-poly(γ-ethyl-l-glutamate) diblock copolymers (mPEG-b-PELG) loaded with combretastatin A4 disodium phosphate (CA4P) and cisplatin (CDDP) was developed by Yu et al. (2019) for the local treatment of colon cancer [87]. The hydrogel showed concentration-dependent thermo-gelling behavior, tunable in vivo biodegradability and controlled drug release properties. In vivo studies performed in C26 tumor bearing mice proved that the formulation possessed the highest antitumor efficacy in comparison with the other experimental groups (animals treated with saline, blank gel, gel loaded with CA4P, gel loaded with CDDP, free CA4P and CDDP).

Lo et al. [88] developed an in situ gelling formulations based on a mixture of a thermo-sensitive poloxamer (F127) and a pH-sensitive polyacrylic acid (PAA) for colon cancer therapy. In particular, liquid and solid dosage forms were developed. F127 and PAA when administered in a liquid form (as polymer solutions) instantly gelify in colorectal physiological condition (pH 7.4 and 37 °C). The solid form, a cocoa butter-based solid suppository with incorporated F127 and PAA mixture, melts and instantly forms a gel. Epirubicin was chosen as a model anticancer drug. The formulations were retained in the upper rectum of rats for at least 12 h and were characterized by a higher relative bioavailability, in comparison with a drug rectal solution.

An innovative in situ gelling system to be applied on the mucosa of the distal colon via rectal route was recently developed by our group [7]. The system was based on three polymers having different functions such as GG, polymer capable of gelling in presence of ions, MC, polymer characterized by a gelation temperature close to 50 °C, and HPC, polymer capable to interact with mucin/mucosa. The three polymers synergistically act to obtain the formation of a protective gel layer with a prolonged permanence on the mucosa. A DoE approach (simplex centroid mixture design) was used to identify the optimal quantitative composition of the formulation. Different response variables were considered: (i) vehicle viscosity at room temperature, (ii) increase in vehicle viscosity on increasing temperature (from room to physiological value) and upon dilution with simulated colonic fluid (SCF), (iii) viscoelastic behavior, (iv) thixotropic area and (v) mucoadhesion properties of the gel. The system was loaded with maqui berry extract (MBE), characterized by antioxidant and anti-inflammatory properties.

Few works have been published on in situ gelling formulations for oral delivery of drugs. In particular, Daia et al. incorporated a Class III drug/permeation enhancer complex into an in situ gelling vehicle, represented by Cremophor [89]. The aim was to minimize the dilution effect of the formulations in the gastrointestinal lumen and to synchronize the diffusion of both a drug and a permeation enhancer to the duodenum that was the absorption site. The authors proved that in situ gelling formulations produced a significantly higher bioavailability of the tested drug when orally administered in rats in comparison with non-gelling PEG 400 based vehicle.

Other authors developed a modified in situ gelling ALG formulation for the sustained release of dextromethorphan (DX) in the gastrointestinal tract. A micronized solid matrix based on a mixture of DX and Eudragit S 100 was dispersed in an ALG solution (2% *w*/*w*). ALG vehicle was responsible for the formation of a gel in presence of the gastric medium [90].

## 6. Vaginal Route

The vagina is a suitable route for the administration of drugs with local and systemic effects [3]. Antimicrobials, hormones, spermicides and anti-inflammatory drugs have been traditionally locally administered via vaginal route [91]. Moreover, the vagina shows several features, such as a rich blood supply, a large superficial area and the possibility of bypassing first pass metabolism, which make vaginal route suitable for the systemic administration of drugs, e.g., calcitonin [3].

On the other hand, the self-cleaning action of the vaginal fluid is responsible for the leakage of many dosage forms with a consequent decrease of the therapeutic effects [3].

The employment of mucoadhesive and in situ gelling formulations represents the strategy proposed in the last decades to increase the residence time of liquid vaginal formulations at the action/absorption site. In the last 10 years, two approaches have been proposed to obtain an increase in formulation viscosity upon administration, whereby the employment of mixtures of (i) thermo-sensitive polymers and (ii) mucoadhesive polymers able to interact with proteins present in the vaginal cavity, forming weak bonds (e.g., hydrogen bonds) or disulphide bonds with proteins containing thiol groups.

Baloglu et al. [92] and Ibrahim et al. [93] developed in situ vaginal thermo-responsive mucoadhesive gels containing econazole and metronidazole, respectively. These consisted of mixtures of different poloxamers. The formulations showed non-Newtonian flow at room temperature and were characterized by a step increase in viscosity, at body temperature.

More recently, Rocha de Araújo et al. developed an in situ gelling liquid crystalline precursor system consisting of 30% of oleic acid and cholesterol (7:1), 40% of ethoxylated and propoxylated cetyl alcohol and 30% of a dispersion of 16% P407 [91]. The authors investigated the effect of the dilution with simulated vaginal fluid (SVF) on formulation behavior by means of different techniques, such as polarized light microscopy (PLM), small-angle X-ray scattering (SAXS), rheological and texture profile (TPA) analysis and mucoadhesion measurements. Moreover, an in vitro drug release test, using hypericin (HYP) as the drug model, and cytotoxicity assay were performed. The diluted formulation showed anisotropy and a non-Newtonian viscous behavior. The authors proved that the dilution with SVF led to the formation of a more viscous and long-range ordered system able to adhere to the vaginal mucosa. Moreover, the formulation was characterized by a HYP sustained release profile and was biocompatible toward L-929 cell line.

Our group compared the potentiality of P407/chitosan lactate (CS-L) and CS-L/GP mixtures as mucoadhesive thermally sensitive vehicles for the treatment of vaginal mucositis [94]. In particular, the addition of CS-L to P407 was responsible for an increase in P407 gelation temperature from 30 °C to the physiological temperature. While the dilution of P407/CS-L mixture with SVF produced an increase in gelation time, no variation of such parameter was observed in the case of CS-L/GP mixture. CS-L/GP mixture was characterized by higher mucoadhesion and bioactive properties than P407-based mixture.

**Figure 5:**
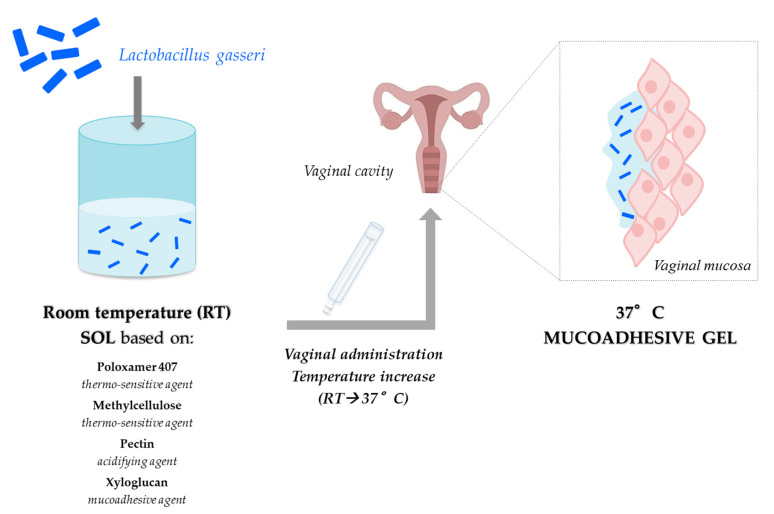
Rationale for the development of a vaginal formulation intended for the treatment of candidosis recurrences: An increase in temperature (from room to body temperature) is responsible for the transition of a polymeric solution loaded with *Lactobacillus gasseri* into a mucoadhesive gel after vaginal administration [95] (MDPI, 2019).

Recently, we developed a vaginal formulation for the delivery of *Lactobacillus gasseri* as prevention strategy against candidosis recurrences [95]. In particular, it consisted of a mixture of P407 and MC, which act as thermo-sensitive polymers, as well as PEC and xyloglucan (XYL), which were used as a mucoadhesive agent. The association of P407 (15% *w*/*w*) with MC (1.5% *w*/*w*) was responsible for an increase in gelation extent at 37 °C, while the presence of 0.5% *w*/*w* PEC produced a reduction of vehicle pH and viscosity at 25 °C, functional to an ease administration. The addition of XYL (0.25% *w*/*w*) produced an increase in the formulation mucoadhesive properties and a modulation of the gelation temperature (Figure 5).

Last year, Jalil and colleagues synthetized a novel polymeric excipient by thiolation of GG that was identified as a suitable polymer for the preparation of mucoadhesive films for the treatment of vaginal microbial infections due to its in situ gelling and film forming properties; in particular, GG was conjugated with 2-(2-Amino ethyldisulfanyl) nicotinic acid (AMENA) via amide bond formation. S-protected GG-based films, prepared by casting, were loaded with metronidazole, demonstrating the capability to provide a sustained drug release [96].

A mucoadhesive tetradeca-thiolated β-cyclodextrin (β-CD)was recently synthesized by Asim et al. [97]: all primary and secondary -OH groups at C-6 and C-2 position were substituted with SH groups. Rheological studies were performed to investigate the viscoelastic behavior of the modified oligomer, using porcine intestinal mucus and fibrous structural protein keratin. The addition of 0.5 and 2% (*w*/*v*) tetradeca-thiolated β-CD to mucus and keratin was responsible for an increase of the dynamic viscosity up to 7.6- and 5.9-fold, respectively, within 4 h at 37 °C [97].

Other in situ gelling formulations are solid formulations that absorb the vaginal fluid forming a gel able to adhere to the vaginal mucosa and to release in a prolonged way the drugs loaded.

Aboud et al. developed cubosomal in situ gelling sponges (CIS) for uterine targeting of sildenafil citrate (SIL), a type 5-specific phosphodiesterase inhibitor employed for the management of infertility in women [98]. Cubosomal dispersions, composed by glyceryl monooleate as lipid phase, P407 as surfactant and PVA as stabilizer, were prepared by an emulsification method. After incorporation of CS (2% *w*/*w*), small sponges were obtained by freeze-drying. The efficacy of SIL-loaded cubosomes was compared to that of intravaginal free SIL sponges (FIS) and oral SIL solution after administration in Wistar rats. SIL-loaded cubosomes were characterized by a drug sustained release over 8 h. A significant enlargement in endometrial thickness with congestion and dilatation of endometrial blood vessels was observed after intravaginal CIS administration [98].

Other authors developed a vaginal insert containing the probiotic strain *Lactobacillus crispatus*, the prebiotic substrate fructo-oligosaccharide and the antioxidant agent ascorbic acid, intended for the prophylaxis and therapy of vaginal infections. The inserts were based on HPMC and were prepared by freeze-drying. They were mucoadhesive and able to modulate lactobacilli release, depending on the different amounts of fructo-oligosaccharide [99].

## 7. Intravesical Route

In situ gelling formulations have aroused a certain interest as ideal localized and sustained delivery systems for chemotherapeutics. After intra- or peritumoral injection, such systems, in a sol-state before administration, turn into hydrogels in response to certain stimuli and locally release the drug in a controlled manner. The increase in drug concentration at the target site (tumor) maximizes the anticancer activity and, at the same time, minimizes the systemic toxicity; moreover, the hydrogel three-dimensional structure ensures a sustained drug release that prolongs the tumor exposure time to the chemotherapeutic agent [100,101]. In particular, a deep review of the literature suggested that a certain number of published papers were focused on the use of in situ gelling systems in the local treatment of bladder cancer.

Bladder cancer is one of the most common malignancies affecting the urinary tract, in particular the inner urothelial lining of the bladder. Nowadays, the gold standard treatment involves the surgical transurethral resection, followed by the intravesical instillation of chemotherapeutics to prevent tumor recurrence and/or progression. The post-operative treatment, consisting in the administration of drug solutions directly into the bladder via a catheter, guarantees high drug concentrations at the site of damage, thus reducing systemic toxicities. Nevertheless, the effectiveness of the instilled chemotherapeutics could be impaired by both the limited urothelium permeability that prevents drug diffusion into the cancerous tissue and the short drug residence time in the bladder. In fact, after local administration, drug solutions undergo to dilution in the patient urine and, approximately every 2 h, are largely washed out during bladder voiding. Such limitations require multiple drug instillations during the day, which could lead to bladder fibrosis, risks of infections and patient discomforts [102,103,104].

The use of composite drug delivery systems, in particular in situ gelling formulations loaded with nanoparticles, appears as a valuable strategy to (i) improve the solubility of lipophilic drugs (i.e., paclitaxel), (ii) increase the permeability through the urothelium of hydrophilic compounds and, finally, (iii) prolong drug residence time in the bladder [104].

Recently, Men and colleagues proposed the encapsulation of deguenil (D), a chemoprotective and therapeutic hydrophobic agent, in novel cationic nanoparticles, composed of *N*-[1-(2,3-dioleoyloxy)propyl]-*N*,*N*,*N*-trimethylammonium methyl sulfate (DOTAP) and monomethoxyl poly(ethylene glycol)-poly(ε-caprolactone) (MPEG-PCL) (D/DMP), loaded in a Pluronic F127-based thermo-sensitive formulation (F). The developed D/DMP-F composite system proved to be a good candidate for the treatment of bladder cancer, acting as a drug depot for sustained deguenil release. The Pluronic F127 formulation, in a liquid state, at room temperature, could be easily instilled through a catheter in the bladder, where it becomes a gel due to the increase in temperature within 10 min. The incorporation in the hydrogel of D/DMP cationic nanoparticles, which were responsible for an improvement of both drug solubility and uptake by bladder cancer cells, prevented their rapid elimination during the first urination, thus increasing drug exposure time [105].

However, in situ gelling systems, such as that developed by Men et al., may not be completely safe, as a result of their high viscosity and low mucoadhesive behavior; in fact, after the sol–gel transition, the hydrogels can easily detach from the bladder wall, thus causing a urinary tract obstruction.

**Figure 6:**
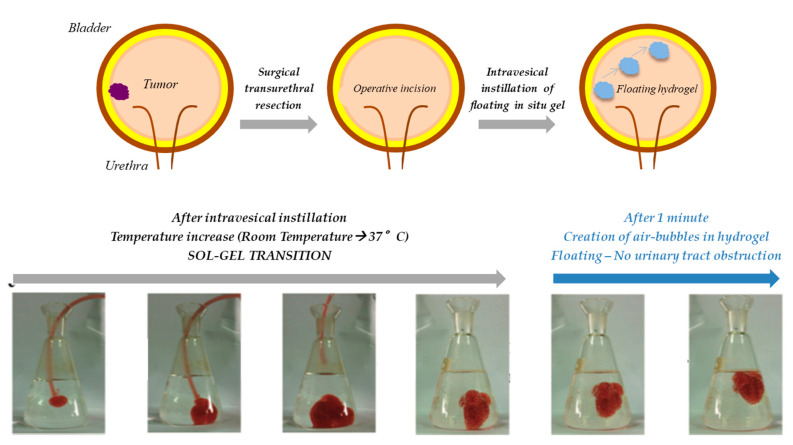
Schematic representation of the management procedure for the treatment of bladder cancer; it involves the surgical transurethral resection, followed by the intravesical instillation of chemotherapeutic-loaded floating in situ gel, using P407 as thermo-reversible agent. The strategy proposed by Lin and co-workers aimed to avoid the obstruction of the urinary tract (Adapted with permission from [106], ELSEVIER, 2014).

In an attempt to overcome this limitation, in the following years, the research group of Lin developed three different floating hydrogel delivery systems, always using P407 as thermo-reversible agent (Figure 6).

In the first work, the authors designed an in situ gelling system, composed of P407 and NaHCO_3_, in which adriamycin-loaded human serum albumin (ADR-HSA) nanoparticles were dispersed. The system could be easily administered in the bladder, through a catheter: It was liquid before instillation and turned into a gel at body temperature. In the acidified urine, the presence of H^+^ caused the NaHCO_3_ decomposition and the production of CO_2_ micro-bubbles within the three-dimensional structure of the hydrogel, which are responsible for the system floating in urine: ADR could be released in a controlled manner and the risk to block the urinary tract was reduced. Nevertheless, the hydrogel floating occurred only in an acid environment (pH < 5.6): The process of urine acidification could be harmful for the patient, who has to ingest some compounds, such as ammonium chloride or vitamin C, with the risk of increasing side effects, including acid–base imbalance and electrolyte disturbances [106]. Therefore, in the second work, NaHCO_3_ was substituted by ammonium bicarbonate (NH_4_HCO_3_), an agent commonly used in food industry to create air bubbles in baked goods. Even if the use of NH_4_HCO_3_ allowed the hydrogel floating in neutral conditions, such a compound is particularly unstable in liquid solution and, thus, could impair the physical stability of the system during storage [107]. Considering also that both NaHCO_3_ and NH_4_HCO_3_ are inorganic substances, which could react with the drug loaded into the hydrogel, the use of these two floating systems seemed to be limited in the clinical practice. Therefore, in 2016, Lin and colleagues developed a floating hydrogel with no additive for micro-bubble production, only exploiting the foamability of P407 that is a non-ionic surfactant: Air-filled micelles consisting in P407 molecules, with the hydrophobic tail facing the gas phase, while the hydrophilic one faces the water, could be obtained by simply shaking of the P407 solution before instillation. The air micro-bubbles formed persisted over a certain period of time in the P407 solution due to its high viscosity also at room temperature; after local administration, the instilled liquid system turned into a gel, entrapping within its structure the micro-bubbles. The drug delivery system developed was recognized as safe. After sol–gel transition, the hydrogel immediately floated in urine, guarantying a controlled drug release [108].

In the same year, Sun and colleagues designed a magnetic thermo-sensitive system, composed of chitosan (CS), GP and Fe_3_O_4_ magnetic nanoparticles (Fe_3_O_4_-MNPs) and containing mitomycin C (MMC) as chemotherapeutic agent [102]. After instillation in the bladder via a catheter, Fe_3_O_4_-MNPs targeted the solution to cancer tissue under the influence of a magnetic field and the gelation process occurred: It is well-known that the increase in temperature (from room to body temperature) induced the physical cross-linking of CS in presence of GP. The authors demonstrated, both in vitro and in vivo, the improved antitumor activity of MMC when loaded in the Fe_3_O_4_-CS/GP formulation with respect to the conventional delivery systems. This result could be attributed to (i) the prolonged retention (more than 72 h) of the system on the bladder wall due to the mucoadhesive potential of CS, (ii) the hydrogel protective effect that improved MMC stability, (iii) the MMC sustained release and (iv) the increased MMC uptake by cancer cells thanks to CS penetration enhancer properties [102].

More recently, GuhaSarkar and co-workers designed a GG-based in situ gelling system containing paclitaxel-loaded liposomes (liposome-in-gel). The low viscosity and the shear-thinning behavior of the system enhanced its instillation into the bladder, where an ion-triggered gelation occurred due to the high ion concentration in urine. The adhesion of the hydrogel formed to the urothelium ensured a localized and sustained paclitaxel release [103].

In Table 1, examples of in situ gelling systems developed in the last decade for ocular, nasal, buccal, vaginal and intravesical administration are reported. Information about administration route, pathology treated, drug loaded, functional polymer, gelation mechanism and major findings obtained is reported.

## 8. Conclusive Remarks

Many papers have been published on the topic in the scientific literature of the last 10 years. The in situ gelling strategy proved to be useful for improving the efficacy of local or systemic drugs administered via non-parenteral routes, prolonging their residence time at the site of action/absorption. The addition of mucoadhesive agents or the employment of polymers having both in situ gelling properties and the capability of interacting with the mucosa/mucus further enhances the formulation performance. In the last year, the approach most widely and successfully employed provides the use in the same formulation of polymers having different mechanisms of in situ gelation to exploit a synergic action between them. Nanotechnology applications in the pharmaceutical/biomedical fields have become a reality today, and in situ gelling formulations have been demonstrated to be a versatile and suitable vehicle for nanosystem administration via non-parenteral route.

## Figures and Tables

**Table 1:** Examples of in situ gelling systems developed in the last decade for ocular, nasal, buccal, vaginal and intravesical administration.

Administration Route	Pathology	Active Pharmaceutical Ingredient	Stimulus	Polymer/s	Major Findings	References
Ocular	Glaucoma	Pilocarpine	Temperature	Gelatin-*graft*-poly(*N*-isopropylacrylamide) (GN) copolymer	Prolonged intracameral high drug concentration (animal model)	[19]
		Pilocarpine	Temperature	Chitosan-*graft*-PNIPAAm (CS-PN) copolymer	Delayed drug release; prolonged antiglaucoma effects (animal model)	[21]
		Brinzolamide	Temperature	Drug-resin (Amberlite IRP-69) dispersed in poloxamer (Pluronic^®^ F-127)/polyacrylic acid (carbopol 934P)	Enhanced drug absorption in the aqueous humor with respect to commercial eye drops (animal model)	[22]
		Betaxolol HCl	Temperature	Poloxamer analogs (P407/P188)/polycarbophil	Prolonged drug release; enhanced drug bioavailability and reduced intraocular pressure (in vitro and in vivo studies on animal model)	[23]
		Acetazolamide (AZA)	Ions	Drug-loaded nanoemulsion (NE) incorporated in gellan gum (GG)/xanthan gum	Prolonged drug release; enhanced therapeutic efficacy (animal model)	[24]
		Brinzolamide	Ions	Gellan gum (GG)	Controlled drug release; extended duration of intraocular pressure reduction (animal model)	[25,26]
		Pilocarpine	Ions	Methacrylated gellan gum	Improved mucoadhesion (ex vivo and in vivo studies on animal model)	[27]
Nasal	Allergic rhinitis	Mometasone furoate (MF)	Ions	Gellan gum	Enhanced efficacy with respect to nasal drug suspension (animal model)	[51]
		Dexamethasone 21-phosphate disodium salt	Temperature	Poloxamer/hydroxypropyl methylcellulose/chitosan (CS)	Prolonged nasal residence time; extended drug release profiles (in vitro studies)	[55]
	Migraine	Zolmitriptan and Ketorolac tromethamine	Temperature	Pluronic^®^ F-127/xyloglucan	Enhanced drug bioavailability with respect to oral administration (animal model)	[62]
		Naratriptan HCl	Temperature	Poloxamer (P407)/carbopol 934P	Sustained drug release, enhanced drug permeation (in vitro and ex vivo studies)	[63]
		Zidovudine (ZVD)	Temperature	Poloxamer	Increased drug permeability; enhanced brain distribution (animal model)	[65]
Buccal	Oral mucositis	Benzydamine hydrochloride	Temperature	Trimethyl chitosan (TMC)/glycerophosphate (GP)	Prolonged drug release; enhanced resistance toward removal physiological mechanisms (in vitro and ex vivo studies)	[70]
		Platelet lysate (PL)	Temperature	Poloxamer 407/sodium alginate	Mucoadhesive properties; cell proliferation properties (wound healing) (in vitro studies)	[71]
		*Hibiscus sabdariffa* extract	Ions	κ-carrageenan (κ-CG)/hydroxypropyl cellulose (HPC)/CaCl_2_	Formulation capability to interact with saliva ions and esophagus mucosa (ex vivo studies)	[73,74]
		Bupivacaine γ-linoleate (Bup-γL)	Temperature	Poloxamer F127/polymers of cross-linked polyacrylic acid (Carbopol^®^ and/or Noveon^®^)	Improved mucoadhesion (ex vivo studies)	[68]
		Benzydamine hydrochloride	Temperature	Poloxamer F127/polyvinylpyrrolidone (PVP)/chitosan	Extended drug release; improved mucoadhesive properties (in vitro and ex vivo studies)	[72]
Gastrointestinal	Gastrointestinal inflammatory diseases	Budesonide (BUD)	Temperature	Poloxamer F-127	Formulation capability to resolve the inflammatory injury in the intestinal mucosa (animal model)	[86]
		Maqui berry extract (MBE)	Ions	Gellan gum (GG)/methylcellulose (MC)/hydroxypropylcellulose (HPC)	Synergic action of the polymers; increased permanence of the vehicle on the mucosa (in vitro studies)	[7]
Vaginal	Vaginosis	Metronidazole	Ions	Thiolated gellan gum (GG) by conjugation with 2-(2-Amino ethyldisulfanyl) nicotinic acid (AMENA)	Improved adhesion on mucosal surface; significant antimicrobial activity; sustained release of metronidazole	[96]
		*Lactobacillus gasseri*	Temperature	Poloxamer 407 (P407)/methylcellulose (MC)/pectin (PEC)/xyloglucan (XYL)	Mucoadhesive properties; capability to preserve *l. gasseri* viability; cytocompatibility (in vitro studies)	[95]
Intravesical	Bladder cancer	Deguenil (D)	Temperature	Drug-loaded *N*-[1-(2,3-dioleoyloxy)propyl]-*N*,*N*,*N*-trimethylammonium methyl sulfate (DOTAP) and monomethoxyl poly(ethylene glycol)-poly(ε-caprolactone) nanoparticles loaded in Pluronic F127	Extended drug residence time; increased drug concentration within the bladder (animal model)	[105]
		Mitomycin C	Temperature	Chitosan (CS)/β-glycerophosphate (GP)/Fe_3_O_4_ magnetic nanoparticles	Sustained drug release; improved drug retention; enhanced antitumor activity compared with free drug solution; enhanced tumor cell apoptosis (in vitro and in vivo studies on animal model)	[102]
		Paclitaxel	Ions	Drug-loaded liposomes in gellan	Enhanced adhesion on the urothelium and increased penetration into the bladder wall; extended drug retention (animal model)	[103]
